# The microbiome–metabolite–vaccine axis: context-dependent modulators of immunogenicity and durability

**DOI:** 10.3389/fimmu.2026.1972642

**Published:** 2026-10-01

**Authors:** Ge Sun, Jingyi Yang, Suzhen Lv, Lijie Yu, Lihui Si

**Affiliations:** 1 Department of Obstetrics and Gynecology, The Second Hospital of Jilin University, Changchun, China; 2 Department of Obstetrics and Gynecology, Lishu County Traditional Chinese Medicine Hospital, Siping, China

**Keywords:** antibody durability, gut microbiome, immune memory, immunometabolism, microbial metabolites, precision vaccination, systems vaccinology, vaccine immunogenicity

## Abstract

Vaccine responses vary markedly between individuals, yet age, genetics, prior antigen exposure, comorbidity, nutrition and vaccine platform explain only part of this heterogeneity. The intestinal microbiome is a candidate modulator through microbial metabolites and parallel non-metabolic signals. Human studies link microbial features to vaccine outcomes, but taxa rarely replicate across geography, age and platform. Small randomised antibiotic studies identify effects of treatment assignment, not necessarily microbiome mediation; infant microbial rescue has been demonstrated in mice rather than infants. Amongst the pathways considered, short-chain fatty acids have the largest mechanistic evidence base, predominantly preclinical, but acetate, propionate and butyrate differ in targets, exposure and direction of effect, and direct human vaccine mediation remains unestablished. Null findings for BNT162b2 in microbiota-depleted mice and for overall antibody responses in an older-adult probiotic trial argue against universal microbiome dependence or probiotic benefit. We organise the evidence around a microbial function–mediator–exposure–immune-checkpoint–endpoint chain, retaining a primary focus on metabolites. Vaccine take, peak antibody, decay and recall are distinguished from clinical protection. We also separate microbiome-modifying probiotics from antigen-delivery platforms. A decision-gated workflow links signature discovery, source and target verification, independent validation, randomised intervention and demonstrated clinical utility. Microbiome-guided vaccination remains a research strategy rather than an established clinical practice.

## 1 Introduction

Interindividual variation in vaccine immunogenicity is clinically consequential. Some recipients generate high neutralising-antibody titres, broad Fc-mediated effector functions, durable memory B cells and polyfunctional T cells, whereas others respond weakly or lose protection rapidly. Established influences include developmental stage, immunosenescence, sex, genetics, prior infection or vaccination, maternal antibody, chronic disease, immunosuppressive treatment, nutritional status and vaccine formulation (1). These variables interact rather than operate independently. In influenza vaccination, pre-existing haemagglutinin-specific antibody and CD4 T-cell levels were inversely associated with subsequent fold increases (2). In inflammatory bowel disease (IBD), infliximab treatment was associated with lower antibody concentrations after one dose of BNT162b2 or ChAdOx1 than vedolizumab treatment (3). For oral rotavirus vaccination, maternal serum and breast-milk antibodies and early microbiota development showed geographically dependent relationships with shedding and seroconversion, whereas measured markers of environmental enteric dysfunction were not significantly associated with either outcome (4). None of these associations should be reassigned to the microbiome without mediator-specific evidence.

The gut microbiome adds a potentially modifiable layer. It is not simply a catalogue of organisms in stool but a community whose gene content and activity are conditioned by diet, drugs, geography, infection, intestinal transit and host physiology. Microorganisms ferment carbohydrates to short-chain fatty acids (SCFAs), transform primary bile acids, metabolise aromatic amino acids, synthesise vitamins and polyamines, and release structural ligands such as flagellin, lipopolysaccharide and peptidoglycan. These products can influence epithelial integrity, dendritic-cell state, B-cell metabolism, plasma-cell differentiation and class switching, and antibody production (5). The microbiome may therefore modulate an intramuscular vaccine without contacting its antigen. Metabolites are the primary focus of this review. Flagellin, lipopolysaccharide, peptidoglycan and cross-reactive antigens are considered separately as non-metabolic comparators: their effects cannot establish metabolite mediation.

This distributed action helps explain why a universal “responder microbiome” has been elusive. Stool composition is an imperfect proxy for small-intestinal communities and local metabolic conditions (6, 7). Taxonomic composition alone may not capture function because different organisms can contribute overlapping metabolic activities and related strains may differ in metabolic capacity (7). The relevant bottleneck also varies by vaccine. A live oral vaccine must reach and replicate in susceptible mucosa; an unadjuvanted parenteral vaccine may depend more on tonic innate stimulation (8); and mRNA–lipid-nanoparticle (LNP), adenoviral-vector and adjuvanted-protein platforms provide distinct delivery and inflammatory signals (9–11). Microbiome dependence may consequently vary by platform, age, immune history and immune phase.

Durability introduces a further distinction. A high antibody concentration at six months can reflect a larger initial peak, a slower decay rate or both. Durable antibody responses involve persistent germinal centres, affinity-matured memory B cells and bone-marrow plasma cells (12–14). Mitochondrial pyruvate import supports long-lived antibody-secreting cells (15); booster recall should be measured separately. Microbiome effects on early immunogenicity therefore cannot be assumed to predict persistence or recall.

Previous reviews have synthesised population- and platform-specific associations and candidate mechanisms (5, 16). The directly overlapping review by Rossouw and colleagues also addresses natural adjuvanticity, metabolites and microbiome-targeted interventions (17). Our contribution is an operational comparison of microbial function, mediator provenance, measured exposure, immune checkpoint and endpoint, rather than a claim that these mechanisms are newly recognised. Population, diet, vaccine platform and immune history are contextual variables within that chain, not competing organising units. We distinguish evidence for altered peak, decay or recall from general immune biology, examine positive and null interventions, and specify the validation required before a signature could inform vaccination decisions. **Figure 1** illustrates the conceptual framework; **Table 1** and **Box 1** translate it into pathway-specific comparisons and a proposed research workflow.

**Figure 1:**
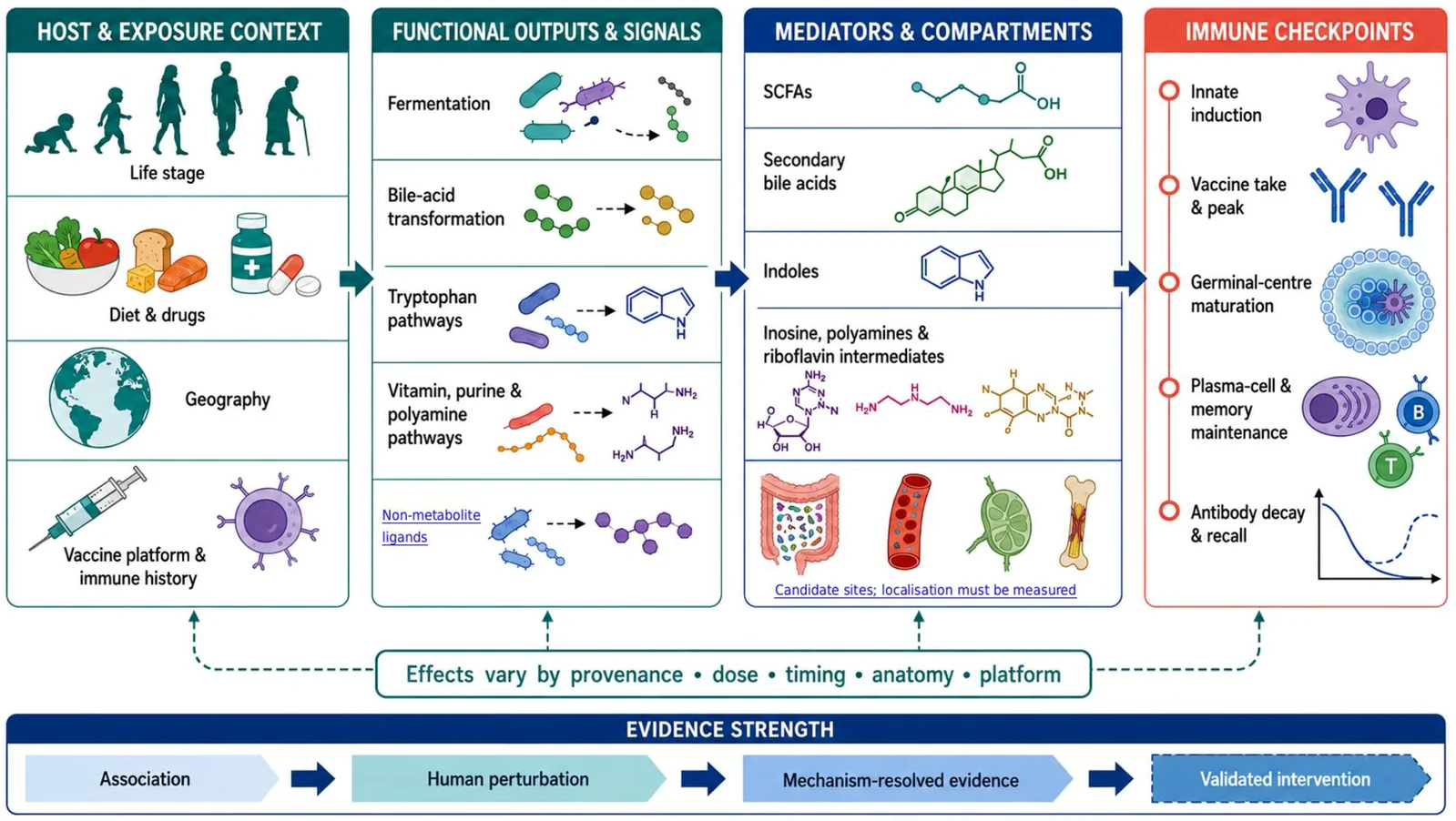
Conceptual framework for the context-dependent microbiome–metabolite–vaccine axis. Metabolic pathways are the primary focus; non-metabolite ligands and antigens provide parallel routes of immune modulation rather than evidence for metabolite mediation. The gut, circulation, lymph node and bone marrow are candidate exposure sites, not a demonstrated transport sequence for every mediator. Inter-compartment arrows have therefore been removed, and localisation must be measured. Host context, dose, timing and vaccine platform condition each proposed relationship. Vaccine take does not establish protection, and late antibody concentration does not by itself establish slower decay. The evidence ribbon denotes requirements to be tested, not a universal causal hierarchy: human perturbation may identify an intervention effect without identifying its mediator, and a validated intervention remains a translational goal. **Figure 1** was prepared using BioRender.com and Microsoft PowerPoint.

**Table 1:** Mediator-specific evidence map for vaccine-relevant metabolic and parallel signalling pathways.

Mediator and provenance	Exposure/principal route	Immune checkpoint and observed effect	Evidence specific to vaccination	Boundary and next discriminating test
Acetate; predominantly microbial fermentation in the intestinal setting	Relatively systemic-accessible SCFA; FFAR2-related signalling and carbon metabolism (7, 49).	Carbon supply and FFAR2 signalling are candidate routes; an acetate-specific vaccine checkpoint remains unresolved (49, 51).	Human SCFA associations are often pooled or contemporaneous; no established acetate-mediated human vaccine effect (35, 46).	Measure acetate separately in the relevant compartment; verify cellular exposure rather than infer production from a taxon.
Propionate; fermentation product	FFAR2/FFAR3 and epigenetic effects, conditional on concentration and cell state (49, 52).	B-cell antibody-related programmes can be suppressed under tested conditions (52).	Mechanistic immunisation/cell evidence; human mediation remains unestablished.	Resolve receptor versus epigenetic effects at physiological exposure and distinguish peak output from persistence.
Butyrate; fermentation and cross-feeding product	Strong local utilisation; FFAR3, HCAR2 and histone-deacetylase-related routes (7, 49, 50, 52).	Supports CD8 memory potential in mice, but can suppress B-cell class switching and plasma-cell differentiation (52, 53).	Animal/cellular effects plus human vaccine-associated SCFA measurements (35, 39); not a universally beneficial mediator.	Quantify local/systemic exposure, cell type and timing; test antigen-specific effects rather than generic inflammation.
Secondary bile acids; host–microbial co-metabolites	Microbial transformation of host bile acids; FXR, GPBAR1/TGR5, VDR and molecule-specific routes (55–57).	Preclinical TH17/Treg and dendritic-cell regulation.	Bile-acid disruption accompanied human antibiotic perturbation, without proof of mediation (41).	Test a chemically defined human-relevant bile acid and target; distinguish drug effects from the proposed mediator.
Indole derivatives; selected microbial tryptophan products	Intestinal AhR signalling and epithelial/IL-22 programmes (58, 59).	Barrier and mucosal immune regulation in non-vaccine models.	No direct human vaccine benefit established by the cited mechanistic studies.	Separate defined indoles from total tryptophan; measure mucosal exposure and a vaccine-specific endpoint.
Kynurenine pathway; predominantly host-derived	Host IDO/TDO activity; AhR and downstream NAD metabolism (60–62).	T-cell proliferation/regulatory differentiation and macrophage metabolic state.	Host immune biology, not proof of microbiome-derived vaccine mediation.	Resolve inflammation, substrate availability and host flux before assigning microbial provenance.
Bacterial sphingolipids; microbial lipid biosynthesis	Strain/gene-level capacity, faecal lipid profiles and bacterial extracts (33).	Cholera-antigen cytokine responses in a human macrophage cell model.	Human memory-B-cell associations plus *in-vitro* perturbation; selected lipidomic subsets were small.	Identify active molecules and relevant exposure; test mediation in vaccinated recipients rather than infer protection.
Inosine, polyamines and riboflavin intermediates; mixed or microbial sources	A2A-receptor signalling, polyamine–hypusine metabolism, or MR1 antigen presentation (63–66).	Context-dependent T-cell function; the routes are distinct rather than a single metabolic class.	Mainly cancer, homeostatic or cellular evidence; no established conventional human vaccine-durability effect.	Treat as separate hypotheses; verify source, target and antigen-specific outcome before translational ranking.
Non-metabolite ligands and antigens; parallel comparison	TLR5, NOD2, innate interferon programmes or antigenic cross-reactivity (8, 68, 69, 72, 73).	Endogenous adjuvanticity and immune conditioning in selected models.	Unadjuvanted influenza models show dependence, whereas BNT162b2 responses were unimpaired in tested microbiota-depleted mice (8, 74).	Do not label these molecules metabolites or assume a shared platform effect; distinguish ligand signalling from metabolic mediation.

AhR, aryl hydrocarbon receptor; FFAR, free fatty acid receptor; FXR, farnesoid X receptor; HCAR2, hydroxycarboxylic acid receptor 2; IDO, indoleamine 2,3-dioxygenase; MR1, MHC class I-related protein 1; NAD, nicotinamide adenine dinucleotide; TDO, tryptophan 2,3-dioxygenase; VDR, vitamin D receptor. Categories describe the evidence actually available, not a formal grading instrument. Entries spanning animal or non-vaccine systems identify plausibility, not human efficacy. The proposed tests are research priorities, not completed validation.

### 1.1 Review approach and analytical framework

This is a targeted narrative review, not a *de novo* systematic review. The 2024 systematic review of 30 publications representing 26 prospective human studies was used as an anchor (16). For this revision, targeted searches of PubMed-indexed records and publisher websites were completed through 8 September 2026, combining microbiome or microbiota with vaccine or vaccination and metabolite, immunogenicity, antibody, durability, antibiotic, probiotic or synbiotic terms. Reference lists and reviewer-nominated studies were also examined. The cutoff denotes completion of this update, not the date of the last included publication. Human longitudinal, perturbation and intervention studies were prioritised; animal and cellular studies were included when they tested a relevant mediator or explained a proposed checkpoint. Null studies were retained. Selection was purposive, without duplicate independent screening or quantitative pooling, so completeness and freedom from selection bias cannot be claimed.

**Table 2** is representative by design rather than exhaustive: it contrasts developmental cohorts, oral and parenteral platforms, randomised positive and null comparisons, metabolite-resolved subsets and late outcomes. Related publications or expanded cohorts are not independent replications. Each study is considered against the same questions: what exposure preceded which endpoint; how many participants contributed to that analysis; was a mediator measured or inferred; how were multiplicity and confounding addressed; and was the finding independently validated? An unreported or unverified safeguard is not assumed. Evidence for antibiotic assignment, a taxon association, a tested metabolite mechanism and clinical utility is kept distinct.

**Table 2:** Illustrative human studies: design, measured endpoint and inferential limits.

Population	Vaccine/platform	Design and timing	Sample size analysed	Measured finding	Evidence boundary
Bangladeshi infants; Huda et al. (18, 19)	BCG, tetanus, hepatitis B and oral poliovirus vaccines	Prospective observational stool profiling at 6, 11 and 15 weeks; immune outcomes at 15 weeks and approximately 2 years.	Initial study: n=48. Follow-up: microbiota n=291; age-2 immune outcomes n=249.	Early Bifidobacterium abundance was associated with selected cellular and antibody responses across routine vaccines.	Temporality and recurrence support a developmental signal, but feeding, infection, nutrition and antibiotic confounding remain; the microbiome exposure was not randomised.
Healthy term infants in the Netherlands; de Koff et al. (20)	PCV10 and meningococcal C conjugate vaccines	Prospective birth cohort with longitudinal 16S profiling and later salivary vaccine-specific IgG.	Cohort n=120; PCV IgG n=101; MenC IgG n=66.	Vaginal delivery and early bifidobacteria or Escherichia coli tracked with selected salivary antibody outcomes.	Delivery mode is a compound exposure; the associations were observational, antigen-specific and measured in saliva rather than serum.
Healthy, vaginally born term infants in Australia; Ryan et al. (22)	PCV13 and Infanrix Hexa (including Hib and diphtheria antigens); PCV13 in mice	Birth-to-15-month observational human cohort; separate germ-free colonisation and defined microbial rescue before mouse PCV13.	Human cohort n=191; antibodies n=160 at 7/15 months, with assay-specific subsets. Mouse n varied by experiment.	Direct neonatal antibiotics were associated with bifidobacterial depletion, inflammatory baseline programmes and lower selected antibodies; defined bifidobacterial or neonatal-probiotic strains restored aspects of PCV13 responsiveness in germ-free mice.	Human antibiotic exposure was observational and microbial rescue was preclinical only; no randomised microbial rescue occurred in infants.
Infants in India, Malawi and the United Kingdom; Parker et al. (4)	Two-dose Rotarix live oral rotavirus vaccine	Method-harmonised prospective cohorts with serial stool, maternal antibody, shedding and infant serum IgA measurements.	n=486: India 307; Malawi 119; UK 60. Endpoint availability varied.	Maternal-antibody and microbiota-development relationships with shedding or seroconversion differed by country and outcome.	Harmonisation supports cross-setting comparison, but site-linked exposures and observational analyses prevent a universal taxonomic rule.
Serotype-3-seronegative Indian infants aged 6–11 months; Grassly et al. (28)	Monovalent type-3 oral poliovirus vaccine	Double-blind randomised three-day azithromycin or placebo trial; vaccination on day 14 and neutralisation on day 35.	n=754 randomised; per-protocol n=705 (348 antibiotic; 357 placebo).	Azithromycin reduced bacterial enteropathogens and selected enteropathy markers but did not improve seroconversion.	Large randomised null result: reducing bacterial burden or selected enteric markers was insufficient to improve immunogenicity.
Infants in Indonesia and Malawi; Wagner et al. (26)	RV3-BB live oral rotavirus vaccine on neonatal or infant schedules	Microbiome analysis nested in trials; specimens collected before and/or after dosing. True pre-dose baseline only in Malawi.	379 infants: Indonesia 193; Malawi 186. 833 stools; endpoint strata smaller.	Higher diversity and Bacteroides were associated with take or shedding in the neonatal schedule, but not consistently with serum IgA or the infant schedule.	Associations were schedule- and endpoint-dependent; most specimens were post-dose, microbiome exposure was not randomised and subgroup testing was extensive.
SARS-CoV-2-naïve adults in Hong Kong; Ng et al. (34)	BNT162b2 mRNA or CoronaVac inactivated vaccine	Prospective, non-randomised platform cohort with baseline and one-month post-dose-2 metagenomics and serology.	n=138: BNT162b2 101; CoronaVac 37. 272 stools.	Baseline candidate taxa associated with neutralising responses differed by platform; B. adolescentis was enriched amongst high CoronaVac responders.	Platform groups differed clinically, associations lacked external validation and internal classification did not establish individual prediction.
SARS-CoV-2-seronegative adults in China; Tang et al. (35)	Two-dose BBIBP-CorV inactivated vaccine	Prospective observational sampling on days 0, 14 and 42; cohort-level metagenomics with targeted faecal and serum SCFAs in a subset.	Cohort n=207; extreme quartiles 52 vs 52; SCFAs n=45 (22 low; 23 high).	Baseline taxa and pathways, and several day-42 SCFAs in the 45-person subset, were associated with day-42 antibody response.	Metabolites were not measured in all 207 participants; baseline faecal SCFAs were not different and contemporaneous day-42 associations do not establish mediation.
Healthy adults with differing pre-existing influenza immunity; Hagan et al. (41)	Seasonal trivalent inactivated influenza vaccine	Two randomised antibiotic comparisons with serial microbiome, metabolome and immune profiling.	Phase 1 n=22 (11 vs 11); phase 2 n=11 (5 antibiotic; 6 control).	Antibodies were unaffected in phase 1; lower H1N1 IgG1, IgA and neutralisation occurred in the 11-person low-pre-immunity cohort.	Randomisation concerns antibiotic assignment, not a microbiome-specific intervention. The responsive phase-2 subgroup was tiny; direct drug effects and mediator confounding were not excluded.
Healthy rabies-vaccine-naïve adults; Feng et al. (43)	Two-dose inactivated rabies vaccine	Randomised five-day antibiotic exposure from day −3; humoral, cellular and B-cell-receptor follow-up to day 180.	18 randomised (9 vs 9); one nonadherent antibiotic participant excluded; analysed 8 vs 9.	The analysed antibiotic-assigned group had lower day-28 rabies IgG and a lower fraction of low- or unmutated class-switched IGHG clones, consistent with reduced naïve-clone recruitment or class switching.	One participant was excluded after randomisation for nonadherence. The reported comparison is not a full intention-to-treat estimate; direct drug effects remain possible, and repertoire features are not lineage tracing.
Healthy Korean adults; Seong et al. (44)	Primary BNT162b2 (n=23) or ChAdOx1 (n=21), followed by BNT162b2 booster	Prospective non-randomised platform cohort with serial microbiota and anti-spike IgG before/after dose 2, at 6 months and after booster.	n=44; stools n=36/39/44/44 by visit; groups split at median half-life or booster rise.	F. prausnitzii was associated with slower anti-spike IgG decay after BNT162b2 and enriched amongst high booster responders.	Small, median-defined strata and non-randomised platforms do not establish that the organism caused slower decay or stronger recall.
Nursing-home residents aged >60 years; Fernández-Ferreiro et al. (47)	BNT162b2 plus Loigolactobacillus coryniformis K8 CECT 5711 or placebo	Randomised supplementation beginning after the first vaccine dose; serum antibodies measured after the second dose.	200 randomised; vaccine immunogenicity assessed in 193 (97 placebo; 96 probiotic).	No significant overall anti-RBD IgG or IgA difference. Positive or suggestive findings concerned selected subgroups.	Overall null result, not a probiotic class effect. Subgroup analyses and intercurrent infection complicate interpretation; metabolite mediation, decay and clinical protection were not established.

BCG, bacille Calmette–Guérin; Hib, Haemophilus influenzae type b; IgA, immunoglobulin A; MenC, meningococcal serogroup C; PCV, pneumococcal conjugate vaccine; RBD, receptor-binding domain; SCFAs, short-chain fatty acids. Selection is purposive as described in Section 1.1. Sample sizes refer to the stated analysis, not automatically to every endpoint. The inference column separates intervention effects from mediator claims; multiplicity control and external validation must be demonstrated for the particular result rather than inferred from study size or design. These reports are not pooled or assigned a formal risk-of-bias score.

Most included evidence concerns bacterial profiles in distal stool, not the virome, mycobiome or archaeome, and not microbial activity at every relevant tissue. Here, vaccine take means evidence that a live mucosal vaccine has established a biological response, often assessed by vaccine-strain replication or shedding; it is not synonymous with protection. Binding antibody, salivary IgG, serum IgA, receptor-blocking surrogate neutralisation, virus-neutralisation assays and cellular responses remain separate endpoints. Target engagement means a measured effect on the intended host receptor, metabolic route or cellular programme at a relevant exposure; a change in stool composition alone does not qualify.

## 2 Human evidence across vaccines and life stages

The aggregate human literature supports context-dependent associations, not a stable taxonomic rule. The 2024 systematic review found at least one association in 26 of 30 publications, but this is a count of reports with any positive result, not a pooled effect, replication rate or estimate of causality. Only five publications enrolled more than 200 participants, and 57% did not adequately identify or address important confounders (16). The genus *Bifidobacterium* recurs in favourable infant associations; broader signals reported at phylum or class level are not directly comparable with genus- or species-level findings. Diversity has no consistent direction. We therefore retain the taxonomic rank and measured endpoint of each result and interpret unreplicated, high-dimensional associations as discovery evidence even when a plausible metabolic function is proposed.

### 2.1 Early life and routine parenteral vaccination

Early life is a sensitive period because the microbiome and immune system mature together. In Bangladeshi infants, early *Bifidobacterium* abundance was associated with stronger cellular or antibody responses to bacille Calmette–Guérin, tetanus, hepatitis B and polio vaccines, including responses detected at two years (18, 19). These studies did not eliminate confounding by breastfeeding, nutrition, antibiotics, infection burden or socioeconomic environment. A European birth cohort linked delivery mode and early communities enriched in bifidobacteria or *Escherichia coli* to selected pneumococcal and meningococcal salivary IgG outcomes (20), but delivery mode also changes maternal exposure, feeding and perinatal care. Salivary IgG should not be treated as an interchangeable measure of serum antibody or clinical protection.

In a US cohort, two-month microbiome evenness was associated with subsequent antibody variability after diphtheria–tetanus–acellular pertussis, *Haemophilus influenzae* type b (Hib) and pneumococcal vaccination; candidate gene-function enrichments were largely nominal, and serum metabolomics was restricted to a 36-infant subset (21). Temporal sampling was a strength, whereas missing outcomes and multiple testing limited inference.

A 2025 study followed 191 healthy term infants from birth to 15 months. Direct neonatal antibiotic exposure was observational and was associated with depleted *Bifidobacterium*, a more inflammatory pre-vaccination transcriptional state and lower antibodies to selected pneumococcal conjugate vaccine, Hib and diphtheria antigens. In germ-free mice, colonisation with a defined *Bifidobacterium* consortium or strains derived from a probiotic used in neonatal units restored aspects of 13-valent pneumococcal conjugate vaccine (PCV13) responsiveness (22). The human component establishes association; microbial reconstitution tests causality only within the mouse model. These complementary results strengthen plausibility without demonstrating a metabolite mediator or successful microbial rescue in infants.

### 2.2 Oral vaccines reveal geography and schedule dependence

Oral vaccines directly encounter the intestinal environment, yet their associations are particularly inconsistent. Rotavirus studies in Ghana and Pakistan identified different, site-specific responder-associated microbial signatures (23, 24). Harmonised cohorts in India, Malawi and the United Kingdom showed site-dependent relationships of maternal antibody and microbiota development with shedding or seroconversion; measured markers of environmental enteric dysfunction were not significantly associated with either outcome (4). Metagenomic reanalysis in India and Malawi associated greater taxonomic and functional diversity with impaired seroconversion (25), whereas a 2025 analysis of neonatal RV3-BB schedules in Indonesia and Malawi associated higher diversity and *Bacteroides* with vaccine take or shedding, but not consistently with serum immunoglobulin A (IgA) or the later infant schedule (26). A 2026 US cohort again found age- and time-dependent associations (27). Collectively, these studies demonstrate ecological context dependence more convincingly than a universal beneficial community. The direction of a shedding association cannot be translated into a direction of protective benefit without a separate immune or clinical endpoint.

Randomised perturbation also yields important null results. Azithromycin reduced bacterial enteropathogens and markers of environmental enteric dysfunction in more than 700 Indian infants but did not improve oral poliovirus vaccine immunogenicity (28). In adults receiving rotavirus vaccine, antibiotics changed the microbiome and vaccine-virus shedding, but the prespecified absolute IgA outcome was negative; high baseline immunity and the adult model constrain extrapolation to infant primary vaccination (29).

In an exploratory oral Ty21a study, multiphasic cellular responders had more diverse and complex longitudinal faecal communities; most differentiating operational taxonomic units belonged to Clostridiales (30). In an oral-cholera-vaccine cohort, greater baseline Clostridiales and lower Enterobacterales abundance were associated with subsequent long-term memory-B-cell responses (31). High-dimensional feature selection and small samples make both findings exploratory. A phase 1 MucoRice–cholera-toxin-B vaccine trial also reported microbiota-associated variation in immunogenicity (32). These studies generate route-specific hypotheses rather than transferable microbial targets.

The pathway framework is illustrated by a subsequent cholera study linking gene-level microbial features in 89 vaccine recipients to O-specific-polysaccharide memory-B-cell responses. Sphingolipid-containing Bacteroides xylanisolvens extracts altered cytokine responses to cholera vaccine antigens in a human macrophage cell model (33). This connects a candidate microbial function to an antigen-related cellular effect, but the small selected lipidomic subsets and *in-vitro* experiments do not establish that sphingolipids improved protection or mediated memory responses in vaccinated people.

### 2.3 Adult parenteral vaccines and metabolite-resolved cohorts

In recipients of BNT162b2 or CoronaVac, baseline microbial profiles were associated with neutralising responses and adverse events, with candidate taxa differing by platform (34). In a 207-participant BBIBP-CorV cohort, metagenomic profiles were analysed at the cohort level, whereas targeted faecal and serum SCFA measurements in a 45-participant subset were associated with day-42 antibody responses (35). Stool and antibody measurements at day 42 make reverse causation and contemporaneous co-variation possible; association does not identify an SCFA mediator.

An expanded six-month follow-up of the Hong Kong BNT162b2/CoronaVac cohort combined baseline metagenomics and metabolomics. Baseline faecal tryptophan-related metabolomic features were associated with six-month surrogate neutralising activity after CoronaVac, but the direction and biological origin of this association remain uncertain and require independent replication (36). After BNT162b2, higher baseline *Bifidobacterium adolescentis*, *Bifidobacterium bifidum* and *Roseburia faecis*, together with higher faecal nicotinic acid and gamma-aminobutyric acid (GABA), were associated with higher six-month surrogate neutralisation; after CoronaVac, higher *Phocaeicola dorei* (formerly *Bacteroides dorei*) and lower *Faecalibacterium prausnitzii* were associated with the corresponding platform-specific outcome (36). These associations lacked external validation, and metabolite provenance remained unresolved.

Other cohorts reinforce heterogeneity. Baseline communities were associated with antibody and CD4 T-cell responses in healthy adults and people living with human immunodeficiency virus (HIV) (37). A selected mRNA-1273 cohort associated the class Clostridia, the family Ruminococcaceae and inferred acetate-production pathways with stronger early IgG (38). A larger BNT162b2 study linked several baseline species, serum butyrate and amino acids to high six-month neutralisation, but extreme-responder metabolomics and wide confidence intervals reduced precision (39). These findings support candidate hypotheses, not clinical prediction.

Relative-abundance profiles can change because another organism rises or falls, and their interpretation can shift materially when absolute microbial load is considered (40). Faecal SCFA concentrations reflect the net effects of production, uptake and transit, whereas circulating concentrations also depend on absorption and clearance (7). Dichotomising responders discards trajectory information and should not substitute for longitudinal modelling. Feature selection requires locked external validation. These constraints explain why discovery models should not be treated as individual risk scores.

### 2.4 Human perturbation and special populations

Randomised antibiotic assignment around seasonal influenza vaccination provides informative intervention evidence. Antibiotics reduced bacterial load and secondary bile-acid metabolism and increased inflammatory programmes. Antibody responses were unaffected in an initial 22-participant cohort, whereas lower H1N1 IgG1, IgA and neutralisation were observed in a second 11-participant cohort selected for low pre-existing immunity (41). The small subgroup limits precision. Randomisation supports inference about antibiotic assignment, but direct drug actions and other downstream changes prevent attribution specifically to microbiome depletion or bile-acid mediation. Observational paediatric data also associate cumulative antibiotic courses with lower vaccine antibody concentrations, although microbiome change and antibiotic indication were not measured (42). Necessary antibiotics should never be withheld to modify vaccination.

A separate primary-vaccination trial enrolled 18 rabies-naïve adults and randomised nine to antibiotics and nine to control. One antibiotic-assigned participant had no detectable metronidazole and was excluded from subsequent between-group analyses. The analysed antibiotic-assigned group had lower day-28 rabies-specific immunoglobulin G (IgG) and a lower fraction of low- or unmutated class-switched immunoglobulin heavy constant gamma (IGHG) lineages, consistent with reduced recruitment and class switching of naïve B-cell clones (43). The repertoire result is not direct lineage tracking. Excluding a participant on a post-randomisation adherence measure means the reported comparison is not a complete intention-to-treat estimate; the very small groups and direct drug effects further limit causal interpretation.

In a 44-participant platform-stratified cohort, *Faecalibacterium prausnitzii* was associated with slower anti-spike IgG decay after BNT162b2 vaccination and was enriched amongst high responders to the subsequent BNT162b2 booster (44). The small strata, median-defined groups and observational design cannot establish that the organism altered decay. In immunosuppressed IBD, microbiota composition and metabolites differed by SARS-CoV-2 antibody response, but intestinal inflammation and anti-tumour necrosis factor therapy are strong alternative explanations (45). Human dietary fibre intake and fibre-fermenting taxa showed a positive but non-significant trend with influenza vaccine responses, largely driven by first-time vaccinees, whereas causal support came from mouse dietary-fibre and SCFA experiments (46).

Applied consistently, the human evidence supports three narrower conclusions: developmental associations are recurrent but not source-resolved; randomised perturbations can alter selected endpoints without identifying a mediator; and late-outcome associations remain largely unvalidated. Null primary outcomes and endpoint-specific exceptions carry the same interpretive weight as positive subgroup findings. **Table 2** reports these distinctions explicitly.

## 3 Metabolic and innate mechanisms

**Table 1** applies the same function–mediator–exposure–checkpoint–endpoint chain to each pathway. It separates measured vaccine-related findings from mechanisms imported from infection, homeostasis or cancer immunotherapy; those sources inform hypotheses but cannot be counted as independent evidence of human vaccine mediation.

### 3.1 Exposure, provenance and compartmentalisation

A pathway gene in stool indicates metabolic potential rather than measured production, host exposure or target engagement; realised metabolite output remains context dependent (7). Faeces, small-intestinal samples and systemic plasma represent different ecological or exposure compartments, and faeces may poorly represent small-intestinal microbial or metabolite patterns (6, 7). Mechanistic studies should connect a defined function to a chemically verified metabolite, an anatomically relevant exposure, a host receptor or metabolic route and a vaccine endpoint. Provenance is also conditional: secondary bile acids are host–microbial co-metabolites; kynurenine is predominantly host-derived; and purines, GABA and nicotinic acid can have mixed dietary, host and microbial origins. Paired functional and metabolite measurements constrain attribution but do not alone prove it (7).

Mucosal compartmentalisation also changes the relevant immune readout. Antigen uptake by microfold cells, local T follicular helper (T_FH_) circuits, IgA class switching and polymeric-immunoglobulin-receptor-mediated transport shape small-intestinal vaccine responses (48). Most human microbiome–vaccine studies reviewed here, however, profile distal stool rather than the immunisation site. Direct sampling shows marked spatial differences and individual-specific temporal dynamics between small-intestinal and faecal communities (6). A stool association may therefore mark diet or transit rather than the organisms or concentrations seen by Peyer’s patches. Oral-vaccine studies should align stool, intestinal or mucosal measurements with shedding, local IgA and systemic serology instead of assuming that one compartment represents the others.

### 3.2 SCFAs connect fermentation to lymphocyte bioenergetics

SCFAs have the largest mechanistic evidence base amongst the pathways discussed, predominantly from preclinical systems, rather than established mediation of human vaccine responses. Acetate, propionate and butyrate are not interchangeable exposures. Receptor-characterisation experiments distinguish acetate-favoured FFAR2 activity, propionate activity at FFAR2 and FFAR3, and butyrate activity at FFAR3; these patterns depend on the assay and receptor context (49). Butyrate also engages HCAR2/GPR109A in colonic immune regulation (50). These receptor studies are not vaccine trials. Propionate and butyrate have prominent histone-deacetylase-related effects in B-cell models, whereas receptor signalling, carbon supply and epigenetic regulation should not be assigned equally to every SCFA (51, 52).

In preclinical infection and homeostatic models and B-cell cultures, SCFAs increased acetyl-CoA availability, glycolysis, oxidative phosphorylation, lipid synthesis and antibody secretion (51). In murine antigen models, butyrate promoted the memory potential of activated CD8 T cells through altered oxidative metabolism (53). However, stool concentration cannot specify exposure in a draining lymph node or bone-marrow niche; a physiologically relevant concentration and a cellular readout are needed before inferring the same mechanism in vaccinated humans.

The effect is not uniformly positive. In murine immunisation models and B-cell cultures, higher butyrate or propionate exposure suppressed activation-induced cytidine deaminase, class-switch recombination, somatic hypermutation and plasma-cell differentiation through epigenetic mechanisms (52). Molecule, concentration, tissue and activation state determine whether receptor signalling, carbon supply or histone-deacetylase inhibition dominates. “More butyrate” is therefore not a defensible universal intervention target.

Antibody production is metabolically demanding. Murine B-cell and limited human-cell experiments showed that activated B cells increase Glut1-dependent glycolysis and mitochondrial respiration, and that this metabolic reprogramming is required for proliferation and antibody production (54). Human-cell assays plus murine genetic experiments showed that long-lived antibody-secreting cells require mitochondrial pyruvate import for survival and durable antibody secretion (15). Neither study was a human vaccine trial. A metabolite may consequently affect the plasmablast burst, germinal-centre selection and plasma-cell maintenance differently. Human trials should measure circulating exposure and immune-cell metabolism rather than infer SCFAs from a presumed producer.

### 3.3 Bile acids regulate inflammatory set points

Intestinal microbes transform host primary bile acids into a diverse secondary pool that can signal through farnesoid X receptor (FXR), G protein–coupled bile acid receptor 1 (GPBAR1, also known as TGR5), vitamin D receptor and other receptor networks (55–57). Defined lithocholic-acid derivatives can suppress T helper 17 (T_H17_) differentiation or promote regulatory T (T_reg_) cells (55). Microbiota-dependent bile-acid pools support colonic RORγt^+^ T_reg_ homeostasis through T_reg_-intrinsic vitamin D receptor signalling (56), whereas isoDCA promotes peripheral T_reg_ generation by reducing dendritic-cell immunostimulatory activity through an FXR-linked mechanism (57).

Direct vaccine evidence is weaker. Antibiotic-treated influenza recipients showed marked disruption of secondary bile acids, but no individual bile acid was shown to mediate the antibody effect (41). The bile-acid mechanisms summarised above are preclinical, and the human perturbation study did not identify an individual species as a vaccine mediator (41, 55–57). Human vaccine studies should therefore quantify relevant bile-acid panels and receptor activity whilst accounting for diet, transit, medication and inflammation.

### 3.4 Host–microbial tryptophan metabolism

Microbial and host tryptophan pathways must be separated. The bacterial tryptophan metabolite indole-3-aldehyde can activate the aryl hydrocarbon receptor and induce interleukin-22-dependent mucosal defence in mice (58); indoleacrylic acid from commensal *Peptostreptococcus* can strengthen barrier-associated programmes and suppress inflammation (59). These observations provide plausibility for oral-vaccine effects but are not direct evidence of improved vaccine take.

By contrast, kynurenine is chiefly produced through host indoleamine 2,3-dioxygenase (IDO) or tryptophan 2,3-dioxygenase. Interferon-γ and CD40 ligand can induce macrophage IDO, deplete tryptophan and arrest T-cell proliferation in human primary-cell systems (60). Kynurenine induced aryl-hydrocarbon-receptor-dependent T_reg_ differentiation in murine *in-vitro* systems (61), whilst downstream host kynurenine-pathway flux can contribute to *de novo* nicotinamide adenine dinucleotide metabolism in macrophages (62). Thus, a kynurenine association may reflect host inflammation, microbiome-regulated host co-metabolism or both; it should not be labelled microbial without source-resolving data.

### 3.5 Emerging metabolic candidates

Microbiome-derived inosine can enhance type 1 helper T (T_H1_) activity under appropriate co-stimulation through the adenosine A2A receptor, but causal evidence comes mainly from cancer immunotherapy (63). Polyamine–hypusine metabolism supports translation, tricarboxylic-acid-cycle wiring and histone acetylation, thereby maintaining helper-T-cell lineage fidelity (64); dietary, host and microbial sources remain difficult to separate. Microbial riboflavin-pathway intermediates are presented by MHC class I-related protein 1 (MR1) to mucosal-associated invariant T (MAIT) cells (65, 66). No study reviewed here directly linked this pathway to conventional human vaccine durability.

### 3.6 Parallel non-metabolic signals and innate conditioning

Flagellin, lipopolysaccharide, peptidoglycan and microbial antigens are not metabolites. They are included because an apparent metabolite association may instead reflect co-varying innate ligands or antigenic cross-reactivity. These alternatives require separate testing and do not strengthen a metabolite-specific causal claim merely by sharing a microbial source.

In preclinical models, structural microbial ligands can provide endogenous adjuvant signals. Toll-like receptor 5 sensing of flagellated commensals was required for optimal antibody responses to unadjuvanted influenza vaccine in mice, with weaker responses in germ-free, antibiotic-treated or receptor-deficient animals (8). Early-life antibiotic dysbiosis impaired several mouse vaccine responses (67); nucleotide-binding oligomerization domain-containing protein 2 (NOD2) recognition of microbiota-derived peptidoglycan was required for cholera-toxin mucosal adjuvanticity (68); and microbiota-induced type I interferons maintained dendritic cells in a poised basal state (69). These effects were not uniform across strongly adjuvanted or live vaccines.

Longer-lived innate conditioning is a possible bridge rather than an established vaccine mediator. Trained immunity describes durable functional reprogramming of innate cells and their progenitors through metabolic and epigenetic changes (70). In humans, bacille Calmette–Guérin vaccination can reprogramme the haematopoietic progenitor compartment and downstream myeloid responses (71). Microbial ligands and metabolites could in principle alter this baseline set point, but direct evidence connecting a defined gut microbial metabolite, bone-marrow conditioning and improved antigen-specific durability after a licenced vaccine remains limited.

Cross-reactive microbial antigens are another non-metabolic alternative. Virus-specific memory-phenotype CD4 T cells recognised environmental microbial peptides, but the influenza-vaccination proof of concept involved only two participants (72). Pre-pandemic SARS-CoV-2-reactive clones also recognised commensal antigens (73). These findings do not establish improved licenced-vaccine efficacy.

## 4 Magnitude, durability and platform dependence

### 4.1 Direct microbiome evidence: peak, late concentration, decay and recall

Most microbiome–vaccine studies measure antibody at one post-vaccination visit, conflating peak magnitude, decay rate and recall. A microbial feature associated with day-180 titre may simply mark a larger day-28 peak. A stronger design estimates participant-specific peak and decay slope and adds avidity, neutralisation breadth, Fc-mediated function, memory B cells, antigen-specific T cells and clinically relevant outcomes.

The direct evidence is narrower than the biological scope of durability. Antibiotic comparisons address selected post-vaccination antibody endpoints (41, 43); the Peng and Ng cohorts primarily associate baseline or measured features with a late neutralisation readout (36, 39). Seong and colleagues instead analysed antibody half-life and post-booster responses, but median-defined observational strata cannot establish that a microbial feature caused slower decay or stronger recall (44). Thus, a late concentration, a modelled decay parameter and a booster increment occupy different positions in the pathway framework. None identifies a metabolite-mediated effect on long-lived plasma-cell survival.

These humoral dimensions are not interchangeable. In SARS-CoV-2-infection-naïve recipients, circulating antibody declined whilst memory B cells increased and broadened between three and six months after mRNA vaccination (75). Longitudinal studies also show that antibody quantity may fall as avidity rises (76), and that binding and Fc-effector functions can have platform-, variant- and function-specific trajectories (77). In a 498-participant observational cohort, Wuhan-D614G pseudovirus-neutralising titres declined between months 1 and 6 after BNT162b2 or mRNA-1273 but increased after Ad26.COV2.S; vaccine choice was not randomised and the Ad26.COV2.S group comprised 62 participants (78). Consequently, “durability” should specify the measured feature rather than treat a late binding titre as a complete phenotype.

### 4.2 Germinal-centre and plasma-cell biology as proposed checkpoints

Human tissue studies locate some of these trajectories. In a 14-person fine-needle-aspiration cohort, spike-specific germinal-centre B cells remained detectable 12 weeks after BNT162b2 dose 2 in 8 of 10 participants sampled at that late visit (12). Small nested clonal analyses across blood, lymph node and bone marrow linked persistent germinal centres to continued somatic hypermutation, affinity-matured memory B cells and bone-marrow plasma cells (13). Varicella-zoster-virus-specific bone-marrow plasma cells were detectable years after childhood vaccination and correlated with circulating antibody, although the evidence was cross-sectional (14). Early marrow detection nevertheless does not guarantee stable entry into a long-lived compartment: newly induced influenza-specific bone-marrow plasma cells declined towards baseline within a year in a 15-donor late-follow-up subset (79), and a 19-participant study found SARS-CoV-2-specific antibody-secreting cells largely absent from the phenotypic long-lived marrow subset after mRNA vaccination (80). These findings are not necessarily discordant: detection of antigen-specific cells in bulk marrow does not establish residence in a phenotypically defined long-lived compartment, and cohorts, sampling intervals and assays differed. These tissue studies define proposed checkpoints, not evidence that a gut metabolite mediated vaccine durability.

The analytical consequence is that a single late titre cannot locate the checkpoint. A participant with a high month-6 titre may have started from a high peak but decayed rapidly, whereas another may have a lower peak and a flatter slope. At minimum, a model should include a pre-vaccination value, a peak visit, two post-peak measurements and a booster visit. Random-slope mixed models can accommodate irregular follow-up; prespecified sensitivity analyses should address breakthrough infection, assay censoring and interval variation. Temporal ordering of intervention, metabolite change, target engagement and outcome is necessary but insufficient for mediation; the additional causal assumptions and sensitivity analyses are addressed in Section 6 (81).

Biological phases should therefore be separated. During the early innate phase, microbial ligands and dendritic-cell state may alter induction (8, 68, 69). During days 7–28, carbon supply may affect plasmablast responses, whereas metabolite-sensitive helper-T-cell pathways provide indirect plausibility for effects on germinal-centre support (51, 52, 54, 55, 64). Later, plasma-cell and memory-population maintenance depends on metabolic support (15) and tissue niches (13, 14, 79). These sources span human tissue studies, preclinical vaccination and non-vaccine mechanism and therefore define testable checkpoints rather than proven microbiome mediation in humans. A metabolite that supports early expansion may not support—and could impair—later selection. Accordingly, these studies nominate where a mediator might act; they should not be counted as demonstrations that microbiome modification has changed that checkpoint in people.

### 4.3 Platform dependence and counterevidence

Platform is an effect modifier, not a nuisance covariate. Platform-mechanism evidence should be distinguished from observational microbiome associations. In humans, the second BNT162b2 dose elicited stronger antiviral and myeloid transcriptional programmes than the first dose (82). In mice, the tested ionisable LNP formulation supplied delivery and interleukin-6-linked adjuvant activity that supported T_FH_ and germinal-centre responses (9). A 14-person human cohort showed MAIT-cell activation after the ChAdOx1 MenB.1 vector vaccine, whilst complementary cell and mouse experiments supported a plasmacytoid-dendritic-cell interferon-α and monocyte cytokine pathway (10). A small, non-randomised cross-cohort systems comparison identified early innate states after AS03-adjuvanted influenza vaccination that differed from those after an unadjuvanted formulation, but antigen and cohort also differed (11). These mechanisms make platform-specific microbiome modulation plausible, but they do not validate the taxa associated with BNT162b2, ChAdOx1 nCoV-19, CoronaVac or BBIBP-CorV in non-randomised cohorts (34–36, 44).

Important counterevidence comes from Norton and colleagues: antibody and CD8 T-cell responses to BNT162b2 were not impaired in germ-free or antibiotic-treated mice under the tested regimens (74). This challenges a universal requirement for microbiota-derived adjuvanticity and contrasts with selected unadjuvanted influenza models (8). It does not establish equivalence across all endpoints, exclude subtler effects, or show that every mRNA vaccine is microbiome-independent in humans. The useful prediction is an interaction between platform, baseline immunity and the particular mediator—not a ranking in which one platform universally bypasses the microbiome.

Platform differences can extend into the germinal-centre phase. In an observational comparison of licenced split-virion and investigational mRNA seasonal influenza vaccines, week-26 germinal centres were detected in 5 of 13 mRNA-vaccine recipients and 0 of 15 split-virion recipients; much smaller clonal subsets supported greater B-cell breadth, whereas haemagglutination-inhibition fold changes did not differ significantly (83). This finding supports platform-specific immune bottlenecks; it does not demonstrate that one platform is universally less microbiome-dependent, because assignment was not randomised, vaccine antigens were not perfectly matched and microbial exposure was not measured.

Baseline immunity is a second interaction. Antibiotic effects were observed for primary rabies vaccination and in influenza recipients with low pre-existing immunity (41, 43). In healthy adults aged approximately 18–48 years, prior-season influenza vaccination was associated with smaller antibody fold rises and weaker CD4/T_FH_ responses, although the comparison was observational (84). Established memory may buffer temporary loss of microbial stimulation, whereas naïve B- and T-cell recruitment may be more sensitive. Trials should stratify by baseline antibody and prior antigen exposure and test intervention-by-platform and intervention-by-immunity interactions.

## 5 Translation: from associations to defined interventions

### 5.1 Defined interventions and mixed human evidence

The observational literature has not established a clinically useful taxon or metabolite threshold (16), and no study reviewed here validated routine stool sequencing, faecal metabolomics or a commercial microbiome score before vaccination. Generic probiotic recommendations are also premature. A systematic review found benefit in only a subset of heterogeneous trials (85), and a meta-analysis of adult influenza studies reported strain- and endpoint-specific serological benefits without establishing clinical protection or durability (86).

A triple-blinded 159-participant trial reported higher anti-receptor-binding-domain (RBD) IgA only in an exploratory subgroup of 17 recipients of two mRNA vaccine doses sampled more than 28 days after dose 2 (10 active and 7 placebo); vaccination-to-sampling intervals were not standardised, and no significant difference in neutralising antibodies was demonstrated (87). This result does not establish a probiotic class effect. Associations with infant bifidobacteria cannot be generalised to any commercial *Bifidobacterium* product, to formula-fed infants or to adults.

A relevant counterweight is the randomised trial by Fernández-Ferreiro and colleagues in 200 older nursing-home residents receiving BNT162b2. Amongst 193 participants with immunogenicity measurements, Loigolactobacillus coryniformis K8 CECT 5711 did not significantly increase overall anti-RBD IgG or IgA (47). Signals in infection- or age-defined subgroups were exploratory and did not establish overall benefit, antibody durability or metabolite mediation. Together with the small exploratory trial above, this favours strain-, population- and endpoint-specific evaluation rather than a generic probiotic recommendation.

A *post hoc* multi-omics analysis of 369 older or diabetic vaccine recipients given SIM01 or placebo linked bifidobacterial enrichment and engraftment to later BNT162b2 surrogate neutralisation; metagenomic and metabolomic subsets comprised 280 and 276 participants, respectively (88). This helps frame a testable resident-community–engraftment–metabolite–endpoint chain. However, associations amongst engraftment, benzoic-acid change and neutralisation do not by themselves identify a causal mediator or demonstrate a slower antibody-decay rate.

Population context is essential. In infants, delivery mode, gestational age, feeding and antibiotic exposure can influence colonisation and vaccination (1, 4, 20, 22). In older adults, frailty, CMV serostatus, baseline inflammatory programmes and T-cell phenotypes have endpoint-specific associations; repeated vaccination may reduce fold rise without reducing adjusted absolute titre or seroprotection (89–93). These factors cannot be assigned a single causal role independently of the study question. Section 6 explains why treating all contextual variables as interchangeable adjustment covariates may itself introduce bias.

A rational intervention must specify the deficient function and verify target engagement. A prebiotic should document substrate utilisation and the expected metabolite change. A strain-defined live biotherapeutic should report identity, dose, viability, engraftment, required substrate and safety. A defined metabolite preparation should have a pharmacokinetic window and receptor or metabolic readout. A preparation of inactivated microorganisms should be evaluated as such, rather than presumed equivalent to a purified metabolite. No controlled human trial identified in the reviewed evidence established faecal microbiota transplantation for vaccine enhancement (16). Clinically indicated antibiotics must not be delayed or withheld.

### 5.2 Probiotic-based vaccine platforms: antigen carriers versus adjuncts

Probiotic-based platforms raise two distinct translational questions. An adjunct given alongside a conventional vaccine aims to change microbial ecology, metabolite availability or innate tone. An antigen-delivery platform instead uses a selected bacterial chassis or bacterial preparation to carry the immunogen. Engineering an antigen-bearing strain does not establish that its benefit is probiotic, that it colonises the recipient, or that a metabolite mediates the response. The relevant comparison must distinguish antigen delivery, structural-ligand adjuvanticity and metabolic effects.

A human example is the randomised phase I/II trial of an oral HPV16 E7-expressing Lacticaseibacillus paracasei-based preparation in 165 patients with HPV16-positive cervical intraepithelial neoplasia grades 2 or 3 (94). The administered preparation was heat-attenuated, not a live colonising probiotic. The study assessed therapeutic lesion regression and antigen-specific cellular responses; it was not a trial of prophylactic infection prevention or microbiome-mediated antibody durability. It demonstrates why a bacterial antigen-carrier strategy belongs in the translational discussion whilst remaining distinct from metabolite supplementation.

For future antigen-carrier platforms, the clinically relevant design question is whether the carrier improves antigen-specific immunity beyond an appropriate antigen comparator, and by which route. Where feasible, antigen-bearing and antigen-free carrier comparisons, matched antigen exposure and viability-defined preparations can separate delivery from generic microbial stimulation. A proposed metabolic contribution additionally requires a verified product, relevant exposure and host target engagement; measuring an increased bacterial abundance or an inflammatory cytokine alone is insufficient. Manufacturing must distinguish antigen identity and consistency from microbial viability, genetic stability and metabolite output. These are proposed evaluation requirements, not evidence that engineered probiotics already provide superior licenced-vaccine responses.

The practical opportunity is therefore selective rather than universal: a strain-defined adjunct might correct a demonstrable functional deficit, whereas a non-replicating bacterial carrier might address mucosal antigen delivery without requiring engraftment. Combining both functions could be investigated, but their effects should be disaggregated before attributing clinical benefit to the microbiome–metabolite axis. Product-specific safety and efficacy must precede any personalised recommendation.

### 5.3 Safety and clinical readiness

Live products and faecal microbiota transplantation (FMT) are not interchangeable low-risk alternatives to defined metabolites. Genomic investigation has linked probiotic organisms to bloodstream infection in intensive-care patients (95). Donor-derived extended-spectrum-β-lactamase-producing Escherichia coli transmission after FMT caused bacteraemia in two recipients, one of whom died (96). These observations require explicit assessment of pathogen transmission, antimicrobial-resistance determinants, product contamination and recipient susceptibility; they do not establish the same risk for every strain or preparation.

Candidate vaccine adjuncts should therefore undergo product-specific identity, purity, viability and resistance assessment, with monitoring appropriate to immunocompromised recipients, disrupted barriers and invasive devices. For engineered live platforms, genetic stability, shedding and potential transfer of mobile genetic elements also require evaluation. Inactivation removes replication but does not by itself establish safety or equivalent immunogenicity. Donor screening cannot make FMT risk-free, and no evidence reviewed here justifies its routine use to enhance vaccination. Clinically indicated antibiotics should not be withheld, and vaccination schedules should not be altered on the basis of an unvalidated microbiome score.

An intervention ladder can reduce unnecessary complexity. Dietary standardisation and a defined substrate are appropriate when the missing function is demonstrably substrate-limited; a strain-defined consortium may be investigated when a relevant functional deficit is demonstrated; a defined metabolite preparation may be preferable when exposure can be dosed and monitored directly; and platform or adjuvant adaptation may be more rational when microbial tonic signalling is unlikely to be the limiting step. Personalising vaccination schedules should be considered only after a reproducible vulnerable window is demonstrated. At every level, the prespecified chain must terminate in a functional vaccine outcome, not merely a change in community composition.

## 6 Limitations and a decision-gated research workflow

### 6.1 Evidence gaps, causal roles and interpretive safeguards

The main limitations are not resolved by adding more omics layers. Most human studies sample distal stool and measure bacterial relative abundance rather than activity at the vaccine-relevant site. Portal, lymph-node and bone-marrow exposures are rarely measured; animal metabolite concentrations, anatomy and vaccine regimens may not transport to humans. Selected responder extremes, small metabolomic subsets, correlated feature testing and reuse of related cohorts can overstate reproducibility. Our purposive narrative selection adds another limitation: the examples identify evidentiary bottlenecks but cannot estimate an unbiased average microbiome effect.

Contextual variables require an explicit causal question. Diet is the exposure in a fibre-intervention trial, a potential confounder in a taxon–antibody association, and sometimes an effect modifier of microbial substrate use. Antibiotic assignment is an exposure, whereas microbiome depletion is a candidate mediator; infection prompting antibiotics can confound observational comparisons. Inflammation measured before exposure may confound an association, whilst inflammation caused by the intervention may lie on the pathway. Delivery mode, feeding and medication use likewise have no fixed role. A prespecified causal diagram should guide adjustment; conditioning indiscriminately on post-treatment variables can block the effect of interest or introduce selection bias.

Temporal ordering is necessary, not sufficient, for mediation. Even in a randomised intervention trial, mediator–outcome associations can remain confounded. Identification depends on assumptions about consistency, positivity, measured confounding and treatment-induced mediator–outcome confounders; measurement error and exposure-dependent selection also matter (81). The estimand and assumptions should be stated, indirect effects accompanied by sensitivity analyses, and mediation distinguished from prediction. Intention-to-treat effects should be primary, with adherence-based analyses identified separately. A statistically significant indirect path is not equivalent to biochemical proof.

### 6.2 From a discovery signature to a clinically useful intervention

**Box 1** sets out a proposed workflow with explicit decision points. Its purpose is to prevent a discovery classifier from being mistaken for a clinical test and to distinguish failure to deliver a mediator from failure of the hypothesised immune mechanism. The downstream decision is not “normalise the microbiome” but whether a defined intervention adds benefit beyond standard vaccination for a specified population and endpoint.

Box 1 **Proposed decision-gated workflow for microbiome-informed vaccine research**

1. Define the decision and causal model. Specify population, vaccine platform, baseline immunity and the target endpoint: take, peak response, decay, recall or disease protection. Prespecify contextual variables and their proposed causal roles. Gate: do not proceed with an undefined outcome or an adjustment set unrelated to the causal question.

2. Discover a function–mediator candidate. Collect pre-vaccination microbial functions and chemically verified metabolites with diet, medication and sampling metadata; distinguish measured activity from taxonomic prediction. Control multiplicity and separate training from evaluation data. Gate: a nominal taxon association alone remains a discovery result.

3. Verify exposure and reproduce the association. Confirm source plausibility, relevant concentration and sampling compartment, then test a locked signature in an independent population. Quantify calibration and added information beyond age, baseline titre and vaccine platform. Gate: failure of replication or absent relevant exposure stops clinical extrapolation.

4. Test mediator-specific target engagement. Link the candidate to a host receptor or metabolic programme and an antigen-specific immune readout. Separate carrier, ligand and metabolic effects. Gate: evidence of altered stool composition without the proposed cellular effect does not validate the mechanism.

5. Evaluate a defined intervention. Randomise against an appropriate comparator, prioritise intention-to-treat analysis, monitor product-specific safety and measure peak, repeated decay time points and recall when relevant. Analyse mediation under explicit assumptions. Gate: target engagement without improved prespecified vaccine outcome is not efficacy.

6. Demonstrate clinical utility before personalisation. Require independent confirmation, acceptable harm and feasibility, and improvement in a validated protection-related or clinical endpoint over standard care. Test whether biomarker-guided allocation outperforms an unguided strategy. Gate: without net clinical benefit, retain the signature as a research tool rather than a reason to change vaccination.

Three falsifiable questions follow. Does a low pre-vaccination functional output identify recipients who benefit from a targeted intervention, rather than merely mark poor health? Does a change in a specified metabolite affect the decay parameter after accounting appropriately for peak response and intercurrent infection? Does the same intervention show a prespecified interaction with vaccine platform or prior immunity? Each question requires a negative-result interpretation: no exposure change questions delivery, no target engagement questions the route, and no functional immune benefit despite engagement questions the proposed checkpoint.

Pre-analytics are part of that causal chain. Because faecal SCFA measurements are sensitive to handling, studies should use rapid freezing or a validated stabilisation approach and, where appropriate, stable-isotope-labelled internal standards; a three-donor study showed that stability over hours was analyte- and donor-dependent (97). Fasting status and time since the last meal should be standardised because they materially alter circulating amino-acid and bile-acid profiles (98, 99). Serum/plasma matrix and processing delays can alter selected metabolites, although most of 151 measured traits were comparatively robust under the tested conditions (100). Stool temperature and storage duration can affect multi-omic measurements and should be harmonised; oxygen exposure had only a marginal effect in one six-donor study (101). For untargeted metabolomics, Metabolomics Quality Assurance and Quality Control Consortium (mQACC) reporting recommendations call for clear documentation of pooled quality-control samples, randomised sample order, predetermined acceptance criteria, and monitoring and reporting of intra- and inter-batch effects (102). Raw sequences, metabolomics quality-control data, analysis code and a complete feature-testing ledger should be deposited.

## 7 Conclusions

The microbiome is a credible, context-dependent candidate modulator of vaccine immunity, but no universal responder community or clinically validated metabolic signature has emerged. The most useful explanatory unit is a microbial function–mediator–exposure–immune-checkpoint–endpoint chain, with metabolites as the principal focus and non-metabolic signals as parallel alternatives. SCFAs have substantial preclinical mechanistic support, but the molecules differ and direct human vaccine mediation remains unestablished. Randomised antibiotic comparisons identify effects of treatment assignment, not necessarily microbiome depletion; infant associations and mouse microbial rescue remain different levels of evidence. Null platform and probiotic findings are integral to this interpretation, not exceptions to be dismissed.

Translation should identify a limiting function, verify its exposure and target, and test a defined intervention against distinct peak, decay, recall and protection outcomes. Antigen-bearing bacterial platforms are delivery strategies, not automatic evidence of metabolic mediation. Without independently replicated functional benefit, acceptable safety and clinical utility, microbiome-guided vaccination remains a research strategy.
